# DEVELOPMENT AND REVISION OF THE FAMILY EMERGENCY PREPARATION PLAN AMONG OLDER ADULTS

**DOI:** 10.1093/geroni/igz038.1992

**Published:** 2019-11-08

**Authors:** Zhen Cong, Zhen Cong, James Craig Keaton, Daan Liang

**Affiliations:** 1 School of Social Work, University of Texas at Arlington, Arlington, Texas, United States; 2 Department Of Civil, Environmental, And Construction Engineering, Texas Tech University, Lubbock, Texas, United States

## Abstract

This study examines factors related to holding, revising, and developing an individual emergency preparation plan among older adults who experienced violent tornadoes. A telephone survey was conducted with 543 respondents approximately one year after two violent tornadoes in 2013 with 276 respondents aged 65 or above. Logistic and multinomial logistic regression showed that education was positively associated with a higher likelihood of having a plan among younger but not older adults. Among those who had a plan before the tornado, older, but not younger, adults who experienced more stress were more likely to report the plan as helpful. More stress and having someone in the household with disability increased the likelihood of revising plans afterward among older adults but not among younger adults. Older adults were less likely to develop a new plan and older, but not younger, adults who reported more stress were more likely to develop a plan.

